# Association between non-barrier modern contraceptive use and condomless sex among HIV-positive female sex workers in Mombasa, Kenya: A prospective cohort analysis

**DOI:** 10.1371/journal.pone.0187444

**Published:** 2017-11-27

**Authors:** Diya Surie, Krista Yuhas, Kate Wilson, Linnet N. Masese, Juma Shafi, John Kinuthia, Walter Jaoko, R. Scott McClelland

**Affiliations:** 1 Department of Medicine, University of Washington, Seattle, United States of America; 2 Department of Global Health, University of Washington, Seattle, United States of America; 3 Institute of Tropical and Infectious Diseases, University of Nairobi, Nairobi, Kenya; 4 Departments of Research and Programs, Reproductive Health, Kenyatta National Hospital, Nairobi, Kenya; 5 Kenya AIDS Vaccine Initiative Institute, University of Nairobi, Nairobi, Kenya; 6 Department of Epidemiology, University of Washington, Seattle, United States of America; Massachusetts General Hospital, UNITED STATES

## Abstract

**Background:**

As access to antiretroviral therapy in sub-Saharan Africa continues to expand, more women with HIV can expect to survive through their reproductive years. Modern contraceptives can help women choose the timing and spacing of childbearing. However, concerns remain that women with HIV who use non-barrier forms of modern contraception may engage in more condomless sex because of their decreased risk of unintended pregnancy. We examined whether non-barrier modern contraceptive use by HIV-positive female sex workers was associated with increased frequency of recent condomless sex, measured by detection of prostate-specific antigen (PSA) in vaginal secretions.

**Methods:**

Women who were HIV-positive and reported transactional sex were included in this analysis. Pregnant and post-menopausal follow-up time was excluded, as were visits at which women reported trying to get pregnant. At enrollment and quarterly follow-up visits, a pelvic speculum examination with collection of vaginal secretions was conducted for detection of PSA. In addition, women completed a structured face-to-face interview about their current contraceptive methods and sexual risk behavior at enrollment and monthly follow-up visits. Log-binomial generalized estimating equations regression was used to test for associations between non-barrier modern contraceptive use and detection of PSA in vaginal secretions and self-reported condomless sex. Data from October 2012 through September 2014 were included in this analysis.

**Results:**

Overall, 314 women contributed 1,583 quarterly examination visits. There was minimal difference in PSA detection at contraceptive-exposed versus contraceptive-unexposed visits (adjusted relative risk [aRR] 1.28, 95% confidence interval [95% CI] 0.93–1.76). There was a higher rate of self-reported condomless sex at visits where women reported using modern contraceptives, but this difference was not statistically significant after adjustment for potential confounding factors (aRR 1.59, 95% CI 0.98–2.58).

**Conclusion:**

Non-barrier methods of modern contraception were not associated with increased risk of objective evidence of condomless sex.

## Introduction

As a result of the global antiretroviral therapy (ART) rollout, millions of women living with HIV can expect to survive through their reproductive years [1, 2]. Access to family planning services should be considered an important component of comprehensive HIV care for this population [3]. An analysis published early during the global ART rollout estimated that over 160,000 HIV-positive births could be prevented annually in sub-Saharan Africa if all women in the region had access to contraceptive services [4]. While the risk of vertical transmission of HIV is now recognized to be greatly reduced by ART [5], access to family planning services remains important as a measure to avoid unwanted pregnancy and to choose the timing and spacing of childbearing.

Female sex workers (FSWs) continue to be an important contributor to HIV transmission in Africa [6, 7]. Dual contraceptive method use, conceptualized as condoms to protect against HIV, STIs, and pregnancy plus a non-barrier modern contraceptive method to further reduce the risk of unintended pregnancy, has been recommended for FSWs [8]. Despite this recommendation, concerns remain that HIV-positive FSWs who use non-barrier contraceptives will adopt risk compensation behavior, engaging in a higher rate of condomless intercourse because of their decreased risk of unintended pregnancy. If true, this would increase their risk for acquisition and transmission of STIs even if their HIV infection were effectively controlled by ART. Several studies have found higher rates of condomless sex in women using non-barrier contraceptives [9–11]. However, these behaviors are likely to be context-specific, and not all studies have identified risk compensation behavior in women using non-barrier contraceptives [12, 13]. A broad database across regions and risk groups will be essential for providing a comprehensive understanding of how risk behavior may be influenced by the use of non-barrier contraceptives.

This study tested the hypothesis that non-barrier modern contraceptive use would be associated with a higher frequency of condomless sex, measured by detection of prostate specific antigen (PSA) in vaginal secretions, in HIV-positive FSWs in Mombasa, Kenya. In addition, the association between non-barrier contraceptive use and self-reported condomless sex was explored, facilitating an examination of possible differences in the results obtained using a biomarker versus interview data.

## Materials and methods

### Study design, setting, and procedures

Detailed methods for this prospective cohort study entitled, “Women’s Lifecourse Events and HIV Transmission Potential,” have been published previously [14]. Briefly, enrollment began in October 2012. Participants were recruited from the Mombasa Cohort, a long-term study of FSWs established in 1993 [15]. Women were included in this analysis if they had laboratory-confirmed HIV, were ≥16 years old, and reported exchanging sex for cash or in-kind payments at enrollment. Pregnant and post-menopausal follow-up time was excluded from this analysis. Additionally, visits at which women reported trying to get pregnant (fertility intent) were excluded from this analysis, as intentional non-use of contraceptives and engagement in condomless sex is expected in such circumstances. The protocol was approved by Human Subject Research Committees at Kenyatta National Hospital and the University of Washington. All participants provided written informed consent.

At enrollment, women completed a standardized face-to-face interview to collect demographic information, medical history, contraceptive methods, and sexual risk behavior. A study clinician performed a pelvic examination with collection of specimens for detection of PSA and STIs. Women were informed that vaginal specimens would be tested for STIs. No additional information was provided about the PSA test or its interpretation. At monthly follow-up visits, women were interviewed to determine current contraception methods and sexual risk behavior. All interviews were conducted using standardized interview guides (S1–S3 Files). Physical examination with specimen collection was conducted quarterly. Free outpatient care was provided at the research clinic, including risk reduction education, free condoms with instruction on their correct use, and STI screening and treatment. Women were also provided with ART according to Kenyan National Guidelines, which stated eligibility for ART at CD4^+^ count ≤350 cells per cubic millimeter (or AIDS defining illness) from October 2012 through June 2014, and increased to CD4^+^ count ≤500 cells per cubic millimeter (or AIDS defining illness) from June 2014 through the remainder of our study period (September 2014) [16, 17].

### Measures

The primary outcome in this analysis was condomless sex, measured through detection of PSA in vaginal secretions, a biological marker of recent exposure to semen. Vaginal specimens were collected at enrollment and quarterly visits, and analyzed for the presence of PSA by chromatographic immunoassay (ABAcard, West Hills, California, USA). This test is most sensitive for detecting exposure to semen within the past 24–48 hours [18]. Self-reported sexual risk behaviors including condomless sex were evaluated using participants’ responses to questions about sexual risk behaviors in the past week. These questions were asked at enrollment and monthly follow-up visits. Women were first asked about the total number of sex acts in the previous week. If none were reported, these women were considered abstinent. For the subset of women who reported sex acts in the previous week, the interviewer asked about the total number of sex acts with a condom. If the number of times women had sex was greater than the number of times they had sex with a condom, women were considered to have had self-reported condomless sex. In the subset of women reporting sex in the previous week, 100% condom use was defined as having the number of sex acts with a condom equal to the total number of sex acts. Women were also asked the number of sex partners in the previous week. This series of self-reported behaviors has been used extensively in this population, and measurement of condomless sex using this approach has been associated with biological outcomes including detection of sperm in genital secretions, pregnancy, sexually transmitted infections, and HIV acquisition [19, 20].

The primary exposure was use of any non-barrier modern contraceptive, which was assessed at enrollment and during monthly interviews. Non-barrier contraceptive methods in this population included depot-medroxyprogesterone acetate, progestogen-containing implants, combination oral contraceptive pills, intrauterine devices, tubal ligation, and hysterectomy.

Covariate data were collected at enrollment, then at varying intervals depending on the variable. Sociodemographic variables collected at baseline included age, education, marital status, age at first sex, and number of years in sex work. Marital status was updated annually. Women were considered to be post-partum for nine months following delivery. Fertility intent (“are you trying to become pregnant?”) and fertility desire (“do you want to have any/more children?”) were reported at quarterly visits. Menopause status was evaluated annually using a clinical algorithm. Alcohol use was assessed annually by the Alcohol Use Disorders Identification Test (AUDIT) [21]. Depressive symptoms were assessed bi-annually using the Patient Health Questionnaire-9 (PHQ-9) [22]. Exposure to intimate partner violence (IPV) was determined annually using 13 questions adapted from the World Health Organization (WHO) survey on violence against women [23].

At enrollment and quarterly examination visits, pregnancy status was assessed by detection of urine beta-human chorionic gonadotropin (Plasmatec Laboratory Products, Dorset, UK) and CD4+ T-cell count was measured by FACSCount (Becton Dickinson, Franklin Lakes, New Jersey, USA). The presence of STIs *(Neisseria gonorrhoeae*, *Chlamydia trachomatis*, or *Trichomonas vaginalis*) was assessed at enrollment by nucleic acid amplification tests (Aptima; Hologic, San Diego, California, USA).

### Analyses

These analyses included data from October 1, 2012 through September 30, 2014. Analyses used log-binomial generalized estimating equations (GEE) regression with working independence correlation structure and robust standard errors to estimate relative risks (RRs) and 95% confidence intervals (CIs) for detection of PSA in vaginal secretions at non-barrier contraceptive exposed versus unexposed quarterly visits. This analytic approach accounts for clustering within participants, since individual women could contribute more than one visit.

Multiple regression analysis was performed to control for potential confounding factors. Age at visit was included in the model as a pre-specified adjustment variable. Additional variables including education, marital status, age at first sex, number of years in sex work, post-partum status, fertility desire, moderate or greater alcohol use (AUDIT ≥7), greater than minimal depressive symptoms (PHQ-9 ≥5), exposure to IPV in the past year [14], number of sex partners, and number of sex acts in the past week were considered for inclusion in the model using a forward selection manual model building approach. As in prior studies, values of covariates collected less frequently than monthly were carried forward from the most recent measurement, imputing the same value at intervening months until the next assessment [14, 24, 25]. First, bivariate regression analyses were conducted with each variable to explore the strength of its association with PSA detection. Variables were entered in the multiple regression model, if they were associated with PSA detection in bivariate analyses (*P* <0.10). Variables were retained in the model if they changed the effect for non-barrier contraception as a risk factor for PSA detection by at least 10% on the relative risk scale. This approach was used to limit the model to variables that adjusted for a meaningful level of confounding. A similar model building approach was used to evaluate the association between non-barrier contraception and secondary outcomes including self-reported behaviors. All analyses were conducted in STATA version 13.0.

## Results

A total of 314 women contributed 3,514 monthly visits to this analysis, of which 1,583 were quarterly examination visits.

For the primary outcome, PSA detection, the median number of quarterly visits per participant was 5 (interquartile range [IQR] 2–8) with a median interval of 88 days (IQR 79–98) between visits. This analysis included 332.7 person-years of observation. For secondary outcomes based on self-reported behavior, the median number of monthly visits per participant was 10 (IQR 4–19).

Baseline characteristics of the study sample are shown in Table 1. The median age was 38 years (IQR 32–42, range 20–57). Most women had less than eight years of education (179, 57%). Their median CD4^+^ count was 455 (IQR 328–618) cells per cubic millimeter, and over half were taking ART (187, 60%). About a third of women reported current use of non-barrier modern contraceptives (106, 34%). Recent condomless sex was detected by PSA in 46 (15%) women, while only 20 (6%) reported condomless sex in the past week.

**Table 1:** Baseline characteristics of 314 HIV-positive female sex workers in Mombasa, Kenya.

Characteristic	n (%) or Median (IQR)
**Sociodemographic characteristics**	
Age	38 (32, 42)
Less than eight years of education	179 (57.0)
Number of years in sex work	9 (5, 14)
**Clinical characteristics**	
Baseline CD4 (N = 313)	455 (328, 618)
Taking ART	187 (59.6)
**Reproductive characteristics**	
Any non-barrier modern contraceptive use	106 (33.8)
Depo-Provera	64 (20.4)
Progestogen-containing implants	16 (5.1)
Oral contraceptive pills	10 (3.2)
Tubal ligation	9 (2.9)
Intrauterine devices	5 (1.6)
Hysterectomy	2 (0.6)
Fertility desire^1^	64 (20.4)
**Biomarkers**	
Semen detection by PSA test	46 (14.7)
Laboratory-confirmed STI (N = 310)	38 (12.3)
**Self-reported sexual risk behavior in past week**^2^	
Condomless intercourse	20 (6.4)
Abstinent	118 (37.6)
100% condom use	176 (89.8)
>1 sex partners	100 (51.0)
>2 sexual encounters	80 (40.8)

^1^Fertility desire was assessed by asking, “Do you want to have any/more children?”

^2^Women were asked how many times they had sex in the previous week, and how many times they had sex with a condom. Women were considered to have condomless sex if the number of times they had sex was greater than the number of times they had sex with a condom. Women were classified as abstinent if they reported no sexual intercourse during the past week. Among those reporting sex in the previous week, 100% condom use was defined as having the total number of sexual contacts with a condom equal to the total number of sexual contacts. Among those reporting sex in the previous week, the number of sex partners and sexual encounters were recorded.

The risk of PSA detection and self-reported sexual behaviors by non-barrier contraceptive status are shown in Table 2. Overall, PSA was detected at 20% (104/523) of visits when women were using non-barrier contraception compared to 15% (163/1060) of visits when women were not using a non-barrier method (RR 1.29, 95% CI 0.93–1.79). This relationship remained similar in the final adjusted model, which included only age (adjusted RR [aRR] 1.28, 95% CI 0.93–1.76).

**Table 2:** Risk of prostate specific antigen detection in vaginal secretions and self-reported sexual behaviors in women using versus not using non-barrier modern methods of contraception.

Outcomes	Visits using non-barrier modern contraception	Visits not using non-barrier modern contraception	Unadjusted		Adjusted	
	n (%)	n (%)	RR (95% CI)	*P*-value	aRR (95% CI)	*P*-value
Semen detection by PSA	104/523 (19.9)	163/1060 (15.4)	1.29 (0.93–1.79)	0.12	1.28^a^ (0.93–1.76)	0.13
Self-reported condomless sex	130/1161 (11.2)	166/2353 (7.1)	1.59 (0.97–2.59)	0.064	1.59^a^ (0.98–2.58)	0.060
Abstinence	492/1161 (42.4)	1097/2353 (46.6)	0.91 (0.74–1.12)	0.38	0.92^a^ (0.75–1.12)	0.41
100% condom use^b^	539/669 (80.6)	1090/1256 (86.8)	0.93 (0.84–1.03)	0.16	0.93^a^ (0.84–1.03)	0.15
>1 sex partner ^b^	223/669 (33.3)	588/1256 (46.8)	0.71 (0.52–0.98)	0.04	0.87^c^ (0.66–1.13)	0.29
>2 sexual encounters ^b^	194/669 (29.0)	477/1256 (38.0)	0.76 (0.55–1.06)	0.10	0.92^c^ (0.70–1.20)	0.53

^a^Adjusted for age;

^b^Analyzed only among 1,925 visits where women reported any sexual activity in the past week,

^c^Adjusted for age, number of years in sex work.

RR, Relative Risk; aRR, adjusted Relative Risk; PSA, prostate-specific antigen.

Denominators represent visits at which outcomes (PSA detection or self-reported sexual risk behaviors) were assessed, and for which responses to questions about non-barrier modern contraceptive use were not missing.

Condomless sex in the past week was reported at 11% (130/1161) of visits using non-barrier methods of contraception compared to 7% (166/2353) of visits not using non-barrier methods (RR 1.59, 95% CI 0.97–2.59). Results were similar after adjustment for age (aRR 1.59, 95% CI 0.98–2.58). Additionally, condomless sex was reported less frequently than it was detected (by PSA test) at visits where women reported using non-barrier methods of contraception (semen detection by PSA, 20% vs. self-reported condomless sex, 11%), and also at visits where women did not report using non-barrier methods of contraception (semen detection by PSA, 15% vs. self-reported condomless sex, 7%).

Women reported having more than one sex partner less frequently at visits where they reported using non-barrier methods of contraception. This result was significant in unadjusted analyses (RR 0.71, 95% CI 0.52–0.98), but not after adjusting for age and number of years in sex work (aRR 0.87, 95% CI 0.66–1.13). There were no significant associations between contraceptive use and other self-reported behaviors in the past week, including abstinence, 100% condom use, and >2 sexual encounters.

Of the 314 women included in the analysis, 216 (68.8%) remained in follow-up at the end of this analysis period. The women lost to follow-up did not differ significantly from those retained in the proportion of visits using modern contraception or the proportion of visits with PSA detected (data not shown). However, the women lost to follow-up had a lower proportion of visits with self-reported condomless sex compared to those retained (21/454 [4.6%] vs. 257/3060 [9.0%], p = 0.018).

## Discussion

In this cohort of Kenyan FSWs, use of non-barrier methods of modern contraception was not associated with a higher risk of biological evidence of recent condomless sex. Comparison of the biomarker data to women’s self-report of condomless sex provides a number of additional and interesting observations. First, it is clear that there is substantial under-reporting of condomless sex overall. Second, the results provide some evidence that underreporting of condomless sex influenced by women’s use of a non-barrier method of contraception. Specifically, women using non-barrier modern contraceptives appear to report condomless sex more frequently than women not using a non-barrier method. This differential reporting could be related to HIV-positive women’s experience of stigma associated with pregnancy [26] [27]. As a result, women living with HIV may be more comfortable reporting condomless sex if they are using an additional non-barrier method of contraception to reduce their risk of pregnancy. Alternatively, women who report condomless sex may over-report their use of non-barrier modern contraceptives. However, this explanation seems less likely, because women were asked about their use of non-barrier modern contraceptives before being asked about self-reported sexual risk behaviors.

These results build on prior studies that explore the effect of non-barrier contraception on self-reported risk behavior in sub-Saharan Africa. Some have observed increased risk, while others have not, suggesting that the relationship between non-barrier contraceptive use and self-reported condomless sex is likely to be context-specific. For example, among FSWs in Nairobi, Kenya, consistency of condom use with clients and nonpaying partners was similar with and without use of other types of contraception (AOR 1.04, 95% CI 0.68–1.59 and AOR 0.94, 95% CI 0.48–1.83, respectively) [13]. By contrast, among eastern and southern African HIV-positive women in serodiscordant partnerships, hormonal contraceptive use was associated with increased likelihood of condomless sex (AOR 1.3, 95% CI 1.1–1.5) [9].

A major strength of this analysis was the use of an objective marker for condomless sex. Prior studies of contraception and condomless sex in FSWs have relied on self-report, which is subject to social desirability and recall biases. Another strength was the large sample size, including 314 women who contributed >330 person-years of follow-up. As such, the study had sufficient power to detect even relatively modest effects of non-barrier contraceptive use on detection of PSA in vaginal secretions.

These results should be interpreted in the context the study’s limitations. First, we did not collect data on the type of sexual partner (new client, regular client, or nonpaying partner) for women in this study. Previous studies have shown that frequency of condomless sex among FSWs varies depending on partner type. Specifically, women who use non-barrier methods of modern contraception may be less likely to use condoms consistently with nonpaying partners compared to clients [11, 12]. Second, repeated evaluations could have modified participants’ subsequent behavior and responses (Hawthorne effect), but it is difficult to know the magnitude and direction of this effect. Third, the biological and self-reported measures of condomless sex in this study assess behaviors over different time periods. Women were asked about self-reported condomless sex in the past week, whereas detection of PSA is most sensitive within 24 hours, and may be detected up to 48 hours after condomless sex [18]. However, despite the shorter period captured by PSA detection compared to self-reported condomless sex, PSA was detected more often than self-reported condomless sex, suggesting substantial underreporting [26]. Fourth, the observational study design limits the ability to establish causal relationships. Nonetheless, observation with careful adjustment for confounding factors may be the best way to address this question, because we are interested in the effect of women’s decisions about use or non-use of non-barrier contraception on their risk behavior. Fifth, these data come from a research cohort of HIV-positive FSWs who received free condoms and ongoing risk reduction education. As such, these findings may be most generalizable to other FSWs receiving risk reduction services.

The availability of contraceptive services should be a key component of comprehensive HIV care, reducing the risks of unwanted pregnancies and vertical transmission of HIV. Data from this study suggest that among HIV-positive Kenyan FSWs receiving free condoms and risk reduction education, the use of non-barrier methods of modern contraception was not associated with a higher risk of objective evidence of condomless sex, but may influence the way women report sexual risk behavior. In settings where women who report using non-barrier contraception are also more likely to report condomless sex, for example in HIV-positive women where pregnancy is stigmatized, the use of a biomarker is likely to provide a less biased estimate of the association between non-barrier contraceptive use and condomless sex. This finding has important implications, as studies that rely solely on self-reported behavior to examine the association between non-barrier contraception and condomless sex may lead to incorrect assumptions, inappropriate allocation of resources, and flawed policy.

## Supporting information

S1 File Mombasa cohort enrollment questionnaire. (PDF)

S2 File Lifecourse enrollment questionnaire. (PDF)

S3 File Lifecourse general monthly follow up questionnaire. (PDF)
