# Supplementary material for: Association between non-barrier modern contraceptive use and condomless sex among HIV-positive female sex workers in Mombasa, Kenya: A prospective cohort analysis
Source: PLoS One. 2017 Nov 27;12(11):e0187444. doi: 10.1371/journal.pone.0187444 (PMC5703462; doi:10.1371/journal.pone.0187444)
Supplement: S1 File — (PDF) [file pone.0187444.s001.pdf]

ENROLLMENT QUESTIONNAIRE

1. Date of visit (day/month/year) ___/___/___

2. Age __ __

3. Nationality 1=Kenyan; 2=Tanzanian; 3=Ugandan; 4=Somali; ___
   5=other, specify ______________________

4. Tribe _____________________________________

5. Place of birth: Town ____
   Province _______________ Country ______________________

6. Religion 1=Protestant; 2=Catholic; 3=Moslem; ___
   4=other, specify ________________________

7. Marital status 0=never married; 1=currently married; 2=widowed/divorced ___

8. Years of education __ __

9. Age at first sex __ __

10. Workplace (1=bar/restaurant/guesthouse; 2=nightclub; 3=home-based; 9=other) ___

11. Years as a prostitute/barmaid (0 if <1 year) __ __

12. Frequency of sexual intercourse per week __ __ __

13. Frequency of sexual intercourse with condoms per week __ __ __

14. Average # of different partners per week __ __

15. Average charge for sex (KSh) (enter 9 if living expenses provided) __ __ __ __

16. Do you have:
    a. Vaginal sex? 0=no; 1=yes ___
    b. Oral sex? 0=no; 1=fellatio; 2=cunnilingus ___
    c. Anal sex? 0=no; 1=yes ___
    d. Vaginal sex during menses? 0=no; 1=yes ___

17. Do you engage in deep kissing or kissing involving the tongue? ___
    If yes,
    a. Frequency of deep kissing per week __ __
    b. Frequency of deep kissing per month __ __
    c. How often do you practice deep kissing when you have sex with clients? __ __ __
       100% (Always); 75% (Often); 50% (Sometimes); 25% (Not often); 0% (Never)
    d. How often do you practice deep kissing when you have sex with your __ __ __
       boyfriend or steady partner?
       100% (Always); 75% (Often); 50% (Sometimes); 25% (Not often); 0% (Never)

18. Current method of contraception (0=none; 1=condoms only; 2=OCP; 3=spermicides; 4=diaphragm; 5=Depo provera; 6=IUD; 7=tubal ligation; 8=hysterectomy; 9=norplant; 10=other [specify __________________________]) ___
    a. If Depo Provera, how long have you used it (months)? __ __
    b. If OCP, how long have you used it (months)? __ __
        Name ___________________________________
        Dose ____________________________________

19. Do you use anything to clean your vagina? If yes, what? 0=no; 1=water alone; 2=with Omo; 3=soap/water; 4=Dettol; 5=other, specify _________________ ___

20. How do you clean inside your vagina? (0=not done; 1=finger; 2=douche bag; 3=bathing flannel; 4=other, specify _____________________________ ___

21. Do you use agents to lubricate your vagina for sex? (0=no; 1=water; 2= saliva; 3=vaseline; 4=KY jelly; 5=other, specify ____________________________ ___

22. Do you put anything into your vagina? (i.e. herbs, traditional cures, drying agents, etc.) ___ 0=no; 1=yes, specify _____________________________

23. Currently pregnant? 0=no; 1-9=estimated month of gestation if pregnant ___

24. Number of pregnancies, including current one if pregnant __ __

25. Number of live births __ __

26. Circumcised? 0=no; 1=yes ___
    If yes,
    a. At what age? __ __
    b. What was removed? 1=clitoris; 2=labia; 3=both ___
    c. Were you sewn up? 0=no; 1=yes ___

27. Do you:
    a. Smoke? If yes, cigarettes per day. __ __
    b. Drink alcohol? If yes, drinks per week __ __
    c. Chew mira? If yes, times per month. __ __
    d. Smoke marijuana? If yes, times per month. __ __
    e. Use cocaine? If yes, times per month. __ __
        If yes, how? 1=sniff; 2=smoke; 3=inject; 4=other, specify______________ ___
    f. Use intravenous drugs? If yes, times per month. __ __

28. In the last 3 months, have you had blood transfusions? 0=no; 1=yes ___

29. Serious medical problems? 0=no; 1=yes ___
    Describe _______________________

30. Have you been hospitalized? 0=no; 1=yes ___
    Describe ________________________________

Interviewer: "Now I am going to ask you some questions about recent sexual activity and vaginal washing."

DURING THE LAST WORKING WEEK:

		Enrollment
31.	# different sex partners	__ __
32.	Frequency of vaginal intercourse	__ __
33.	Frequency of vaginal intercourse with condoms	__ __
34.	Frequency of anal intercourse	__ __
35.	Frequency of anal intercourse with condoms	__ __
36.	Frequency of deep (French) kissing	__ __
37.	Have you used anything to wash the inside of your vagina? *(If no, skip to question 42)*	__
38.	How far inside your vagina did you wash?<br>*0=introitus only (no deeper than fingertip); 1=beyond introitus*	__
39.	What have you used to wash inside your vagina?<br>*0=finger; 1=bathing flannel; 2=other* If other, specify:	__<br>__________
40.	How many times did you wash inside your vagina with...	
	a. Water only?	__
	b. Soap and Water?	__
	c. Antiseptic (e.g. Dettol)?	__
	d. Detergent (e.g. Omo)?	__
	e. Other substance?	__
	If other, specify:	__________
41.	How many hours since you last washed inside your vagina?	__
42.	Have you used a vaginal lubricant for sex? *(if no, skip to 44)*	__
43.	How many times did you lubricate withé	
	a. Water?	__
	b. Saliva?	__
	c. Vaseline?	__
	d. KY Jelly?	__
	e. Other	__
	If other, specify:	__________
44.	Have you put anything in your vagina for purposes other than washing? (e.g herbs, traditional cures, drying agents, etc)?<br>If yes, specify:	__<br>__________
45.	In the last MONTH, how many new sex partners have you had?	__
46.	In the last MONTH, were you forced to have sex without your consent?	__

Initials of the person completing this questionnaire ________
Initials of person entering data in computer ________
Initials of person performing line listing ________
