# Supplementary material for: Association between non-barrier modern contraceptive use and condomless sex among HIV-positive female sex workers in Mombasa, Kenya: A prospective cohort analysis
Source: PLoS One. 2017 Nov 27;12(11):e0187444. doi: 10.1371/journal.pone.0187444 (PMC5703462; doi:10.1371/journal.pone.0187444)
Supplement: S2 File — (PDF) [file pone.0187444.s002.pdf]

LIFECOURSE ENROLLMENT QUESTIONNAIRE

Unless otherwise specified in a question or section text, 0=no; 1=yes

Enter visit number:	**VISIT 0**
1. Date (day/month/year)	___/___/___
2. Urine B-hCG result *0=not pregnant; 1=pregnant*	__
**In this study, we are interested in learning about the health and life experiences of women with HIV. First, I would like to ask you about antiretroviral therapy (ART).**	
3a. Are you <u>currently</u> on ART? ***(If "0=no," skip to Question 4)***	__
b. When did you start ART? (day/month/year) *(This does not include short-term ARVs for PMTCT; e.g. Plan A or Plan B)*	__/__/__
**Now I want you to think about your current or most recent regular male sex partner. This could be a husband or a partner with whom you have (or had) a committed relationship.** *Prompt:* **This is not someone with whom you have had only casual sex or sex in exchange for money or gifts. Some women may not have had a regular partner.**	
4. Do you have a current or most recent regular partner? ***(If "0=no," skip to Question 6)***	__
5. During the last three months, have you had a regular partner?	__
6. During the last three months, have you had a casual partner?	__
7. Current marital status: *0=never married; 1=currently married; 2=widowed/divorced*	__
**Next, I'd like to ask you some questions about pregnancies.**	
8. How many pregnancies have you had, including current one if pregnant? ***(If "0," skip to Question 11)***	__
9. What was the date of your last delivery? (day/month/year)	__/__/__
10. How many live births have you had? ***(Skip to Question 15 if pregnant)***	__
11. Are you trying to become pregnant?	__
12. Do you want to have any/more children?	__
13. How would your current or most recent regular partner feel if you became pregnant? *0=very excited; 1=somewhat excited; 2=would not care; 3=very upset; 4=does not have a regular or current partner*	__
14. Menopausal *(follow menopause clinical decision making tool)*:	__
**Now I would like to ask you some questions about your thoughts and moods. <u>Over the last 2 weeks</u>, how often have you been bothered by the following problems:**	
<u>Responses for questions 15-23:</u>	
0=not at all; 1=several days; 2=more than half the days; 3=nearly every day	
15. Little interest or pleasure doing things?	__
16. Feeling down, depressed, or hopeless?	__
17. Trouble falling or staying asleep, or sleeping too much?	__
18. Feeling tired or having little energy?	__
19. Poor appetite or overeating?	__
20. Feeling bad about yourself- or that you are a failure or have let yourself or your family down?	__
21. Trouble concentrating on things, such as listening to the radio?	__

22. Moving or speaking so slowly that other people could have noticed? Or the opposite- being so fidgety or restless that you have been moving around a lot more than usual? __
23. Thoughts that you would be better off dead, or of hurting yourself in some way? __

    (If "0=not at all" for all questions from 15 through 23, skip to Question 25)

24. If you had any of these problems, how difficult have these problems made it for you to do your work, take care of things at home, or get along with people? __

    0=not difficult at all;1=somewhat difficult; 2=very difficult; 3=extremely difficult

Now I'd like to ask you some questions about your use of tobacco and drugs.

25. Do you:
    a. Smoke? If YES, cigarettes per day. __
    b. Chew miraa, or khat? If YES, times per month. __
    c. Smoke marijuana? If YES, times per month. __
    d. Use cocaine? If YES, times per month. __

       If YES, how? 1=sniff; 2=smoke; 3=inject; 4=other, specify __________ __
    e. Use intravenous drugs? If YES, times per month. __

Now I'd like to ask you some questions about your use of alcoholic beverages <u>during the last year</u>.

26. How often do you have a drink containing alcohol, such as beer, wine, spirits, or locally brewed alcohol (such as changaa, mnazi, busaa, or muratina)? __

    *0=never **(skip to Question 36)**; 1=monthly or less; 2=2 to 4 times a month;3=2 to 3 times a week;4=4 or more times a week*

27. How many drinks containing alcohol do you have on a typical day when you are drinking? One 500mL bottle of beer like a Tusker counts as 1.5 drinks. A glass of wine or locally brewed alcohol counts as one drink. One 'shot' of spirits is one drink. __

    0=1 or 2; 1=3 or 4; 2=5 or 6; 3=7, 8, or 9; 4=10 or more

28. How often do you have 6 or more drinks on one occasion? __

    0=never; 1=less than monthly; 2=monthly; 3=weekly; 4=daily or almost daily

How often <u>during the last year</u> have you:

<u>Responses for questions 29-33:</u>

0=never; 1=less than monthly; 2=monthly; 3=weekly; 4=daily or almost daily

29. Found that you were not able to stop drinking once you had started? __
30. Failed to do what was normally expected from you because of drinking? __
31. Been unable to remember what happened the night before because you had been drinking? __
32. Needed an alcoholic drink first thing in the morning to get yourself going after a night of heavy drinking? __
33. Had a feeling of guilt or remorse after drinking? __
34. Have you or someone else <u>ever</u> been injured as a result of your drinking? __

    0=no; 1=yes, but not in the past year; 2=yes, during the past year

35. Has a relative, friend, doctor, or another health professional <u>ever</u> expressed concern about your drinking or suggested you cut down? __

0=no; 1=yes, but not in the past year; 2=yes, during the past year

Now, I would like to ask you a couple of questions about your HIV test results.

36a. Have you shared your HIV test results with someone? ***(If "0=no," skip to Question 37)*** __

b. With whom? ***Enter number of people in each category:***

__parent	*__boyfriend/partner*	*__sibling*	*__health provider outside this clinic*
__husband	*__other friend/acquaintance*	*__religious leader*	*__other (specify:____________)*
__child	*__employer*		

(If no current or most recent regular partner in Question 4, skip to Question 58)

Next, I would like to ask some questions about your current and past relationships, and how your husband/male partner treats (treated) you. Please answer these questions about the same person you identified as a current or most recent regular male sex partner earlier.

Thinking about your current or most recent regular partner, would you say it is generally true that he:

37. Tries to keep you from seeing your friends? __
38. Tries to restrict contact with your family of birth? __
39. Insists on knowing where you are at all times? __
40. Ignores you and treats you indifferently? __
41. Gets angry if you speak with another man? __
42. Is often suspicious that you are unfaithful? __
43. Expects you to ask his permission before seeking health care for yourself? __

**The next questions are about things that happen to many women and that your current or most recent regular partner may have done to you. Has your current regular or most recent regular partner:** *0=never* ***(skip to next question)****; 1=once; 2=few; 3=many*	**A) <u>Ever?</u>**	**B) <u>In past 12 months?</u>**
44. Insulted you or made you feel bad about yourself?	__	__
45. Belittled or humiliated you in front of other people?	__	__
46. Done things to scare or intimidate you on purpose, e.g. by the way he looked at you, by yelling or smashing things?	__	__
47. Threatened to hurt you or someone you care about?	__	__
48. Slapped you or thrown something at you that could hurt you?	__	__
49. Pushed or shoved you or pulled your hair?	__	__
50. Hit you with his fist or with something else that could hurt?	__	__
51. Kicked you, dragged you, or beaten you up?	__	__
52. Choked or burnt you on purpose?	__	__
53. Threatened to use or actually used a gun, knife, or other weapon against you?	__	__
54. Physically forced you to have sexual intercourse when you did not want to?	__	__
55. Did you have sexual intercourse you did not want to because you were afraid of what he might do?	__	__
56. Did he force you to do something sexual that you found degrading or humiliating?	__	__

(If responses to all Questions 44 – 56 are "0=never," skip to Question 58)

57. Have you been injured as a result of these acts by your current regular partner? __ __

Please think of the acts that we talked about before.

Since the age of 15 years has anyone (other than your current or most recent regular partner):

Responses for questions 58 and 59:
0=never; 1=once or twice; 2=a few times; 3=many times

58. Beaten or physically mistreated you in any way? __ __

59. Forced you to have sex or perform a sexual act when you did not want to? __ __

(If responses to both 58 AND 59 are "0=never," skip to Question 61)

60. Who did this to you? ***Enter 0=no or 1=yes for each response:***

__father/stepfather	*__religious leader*	*__someone at work*	*__boyfriend*
__other male relative	*__police*	*__neighbor/acquaintance*	*__client*
__female relative	*__stranger*	*__teacher*	*__other, specify:____________*

61a. Have you sought help for any violence that you experienced? __

(If "0=no," skip to Question 62)

b. Specify where: ____

62a. Have you ever had sex in exchange for money or gifts? __

b. In the last 12 months, have you had sex in exchange for money or gifts? __

COMMENTS:____

____

____

Initials of person completing questionnaire: __
Initials of person entering data in computer: __
Initials of person performing line listing: __
