# Supplementary material for: Association between non-barrier modern contraceptive use and condomless sex among HIV-positive female sex workers in Mombasa, Kenya: A prospective cohort analysis
Source: PLoS One. 2017 Nov 27;12(11):e0187444. doi: 10.1371/journal.pone.0187444 (PMC5703462; doi:10.1371/journal.pone.0187444)
Supplement: S3 File — (PDF) [file pone.0187444.s003.pdf]

LIFECOURSE GENERAL MONTHLY FOLLOW UP QUESTIONNAIRE

Unless otherwise specified in a question or section text, 0=no, 1=yes

Enter visit number:	**VISIT___**	**VISIT___**	**VISIT___**	**VISIT___**	**VISIT___**	**VISIT___**	**VISIT___**
1. Date (day/month/year)	___/___/___	___/___/___	___/___/___	___/___/___	___/___/___	___/___/___	___/___/___
2. Current method of contraception: *0=none; 1=condoms only; 2=OCP; 3=spermicides; 4=diaphragm; 5=Depo provera; 6=IUD; 7=tubal ligation; 8=hysterectomy; 9=Norplant; 10=other, specify _________*	__	__	__	__	__	__	__
3. Date of last menstrual period (day/month/year)	__/__/__	__/__/__	__/__/__	__/__/__	__/__/__	__/__/__	__/__/__
4. Do you have any serious medical problems? *Describe* ___________________________	__	__	__	__	__	__	__
**<u>During the last week:</u>**							
5. # of different sex partners	__	__	__	__	__	__	__
6. Frequency of vaginal intercourse	__	__	__	__	__	__	__
7. Frequency of vaginal intercourse with condoms	__	__	__	__	__	__	__
8. Frequency of anal intercourse	__	__	__	__	__	__	__
9. Frequency of anal intercourse with condoms	__	__	__	__	__	__	__
**<u>In the last month:</u>**							
10a. Have you had any new sex partners?	__	__	__	__	__	__	__
b. If yes, how many?	__	__	__	__	__	__	__
**<u>General symptoms:</u>**							
11. Fever, constant or intermittent >1month?	__	__	__	__	__	__	__
12. Weight loss	__	__	__	__	__	__	__
13. Night sweats	__	__	__	__	__	__	__
14. Loss of appetite	__	__	__	__	__	__	__
15. Vaginal itching/burning	__	__	__	__	__	__	__
16. Vaginal discharge	__	__	__	__	__	__	__

Enter visit number:	VISIT___	VISIT___	VISIT___	VISIT___	VISIT___	VISIT___	VISIT___
17. Lower abdominal pain	__	__	__	__	__	__	__
18a. Genital sores	__	__	__	__	__	__	__
b. If yes, have they persisted for >1 month?	__	__	__	__	__	__	__
**Skin:**							
19. Itchy skin rash	__	__	__	__	__	__	__
20. Sores in mouth and/or lips for >1 month	__	__	__	__	__	__	__
21. Shingles	__	__	__	__	__	__	__
22a. Other skin conditions (fungus, scabies,…)	__	__	__	__	__	__	__
b. Specify ___________							
**Gastro-intestinal:**							
23. Retrosternal pain when swallowing	__	__	__	__	__	__	__
24. Diarrhea, constant or intermittent for >1 month (≥3 loose stools/day)	__	__	__	__	__	__	__
25. Abdominal discomfort	__	__	__	__	__	__	__
26. Nausea	__	__	__	__	__	__	__
**Neurologic:**							
27. Pain, numbness, or tingling in your hands or feet?	__	__	__	__	__	__	__
**Chest:**							
28. Cough, acute, with or without sputum (<1 month)	__	__	__	__	__	__	__
29. Cough, constant or intermittent for >1 month	__	__	__	__	__	__	__
30. Dyspnea	__	__	__	__	__	__	__
31. Pleuritic chest pain	__	__	__	__	__	__	__
32. Palpitations (extra heart beats)	__	__	__	__	__	__	__
33a. Do/did you receive treatment for tuberculosis?	__	__	__	__	__	__	__
b. If yes, which clinic gives/gave you anti-TB drugs?	___________	___________	___________	___________	___________	___________	___________

Enter visit number:	VISIT___	VISIT___	VISIT___	VISIT___	VISIT___	VISIT___	VISIT___
**Since last visit:**							
34a. Have you visited any other health care provider or facility?	__	__	__	__	__	__	__
If no, STOP here. If yes, complete 34b – d.							
b. If yes, which provider or facility?	________	________	________	________	________	________	________
a. If yes, did you receive any kind of treatment?	________	________	________	________	________	________	________
b. If yes, what treatment? (e.g. name of medicine)	________	________	________	________	________	________	________

COMMENTS:____

____

____

____

Initials of person completing questionnaire:	__	__	__	__	__	__	__
Initials of person entering data in computer:	__	__	__	__	__	__	__
Initials of person performing line listing:	__	__	__	__	__	__	__
